# Advancements in Surface Modification of NiTi Alloys for Orthopedic Implants: Focus on Low-Temperature Glow Discharge Plasma Oxidation Techniques

**DOI:** 10.3390/ijms26031132

**Published:** 2025-01-28

**Authors:** Justyna Witkowska, Jerzy Sobiecki, Tadeusz Wierzchoń

**Affiliations:** Faculty of Materials Science and Engineering, Warsaw University of Technology, 02-507 Warsaw, Poland; jerzy.sobiecki@pw.edu.pl (J.S.); tadeusz.wierzchon@pw.edu.pl (T.W.)

**Keywords:** NiTi shape memory alloys, glow discharge oxidizing, surface engineering, biocompatibility

## Abstract

Nickel–titanium (NiTi) shape memory alloys are promising materials for orthopedic implants due to their unique mechanical properties, including superelasticity and shape memory effect. However, the high nickel content in NiTi alloys raises concerns about biocompatibility and potential cytotoxic effects. This review focuses on the recent advancements in surface modification techniques aimed at enhancing the properties of NiTi alloys for biomedical applications, with particular emphasis on low-temperature glow discharge plasma oxidation methods. The review explores various surface engineering strategies, including oxidation, nitriding, ion implantation, laser treatments, and the deposition of protective coatings. Among these, low-temperature plasma oxidation stands out for its ability to produce uniform, nanocrystalline layers of titanium dioxide (TiO_2_), titanium nitride (TiN), and nitrogen-doped TiO_2_ layers, significantly enhancing corrosion resistance, reducing nickel ion release, and promoting osseointegration. Plasma-assisted oxynitriding processes enable the creation of multifunctional coatings with improved mechanical and biological properties. The applications of modified NiTi alloys in orthopedic implants, including spinal fixation devices, joint prostheses, and fracture fixation systems, are also discussed. Despite these promising advancements, challenges remain in achieving large-scale reproducibility, controlling process parameters, and reducing production costs. Future research directions include integrating bioactive and antibacterial coatings, enhancing surface structuring for controlled biological responses, and expanding clinical validation. Addressing these challenges can unlock the full potential of surface-modified NiTi alloys in advanced orthopedic applications for safer, longer-lasting, and more effective medical implants.

## 1. Introduction

In recent years, increasing demands have been placed on materials intended for medical use, including implants, surgical instruments, and medical devices. Modern medicine increasingly relies on solutions based on the relatively new category of “smart materials”. According to a 2024 report by IMARC Group, the global smart materials market was valued at USD 63.6 billion and is projected to reach USD 142.1 billion by 2033, exhibiting a compound annual growth rate (CAGR) of 8.88% during the forecast period [1]. Among smart materials, shape memory alloys (SMAs) play a significant role, with the near-equiatomic titanium–nickel alloy known as Nitinol being the most prominent example [2,3]. Its unique properties, such as one-way and two-way shape memory effects and superelasticity, enable the design of innovative orthopedic, dental, and cardiovascular implants [4,5,6]. Examples of orthopedic implants utilizing NiTi’s shape memory effects and superelasticity include bone staples, spinal fixation devices, intramedullary nails, orthopedic plates, joint prostheses, and bone scaffolds used in tissue engineering. Due to these properties, the biocompatibility of NiTi alloys has become the focus of numerous research groups worldwide [7,8,9].

The primary challenge in the medical application of NiTi alloys is their high nickel content, which can cause allergic, cytotoxic, and even carcinogenic effects [10,11]. Nickel may be released from the alloy’s surface into the biological environment, posing a health risk. The thin passive oxide layers that spontaneously form on NiTi surfaces offer limited protection, especially for long-term implants. Consequently, various surface engineering methods, including air and water vapor oxidation, electrochemical treatments, laser processing, ion implantation, glow discharge treatments, PVD and CVD techniques, and the deposition of carbon, ceramic, or polymer coatings, have been developed to enhance the corrosion resistance and biocompatibility of NiTi alloys for specific applications [12,13,14,15,16].

Research on the fabrication of NiTi bone implants has shown promising results using low-temperature plasma treatments performed under glow discharge conditions [17]. These processes can be conducted at temperatures up to 300 °C, preserving the alloy’s shape memory effect and superelasticity by preventing the formation of intermetallic phases within the Ni-Ti system beneath the diffusion surface layer [18]. Due to titanium’s strong chemical affinity for atomic oxygen or nitrogen, dense surface layers with nanocrystalline structures and controlled thickness can be produced. The layers, consisting of titanium dioxide (TiO_2_), titanium nitride (TiN), or their mixtures, exhibit biocompatibility, enhance corrosion resistance, and promote desirable biological properties [19,20,21,22]. One notable advantage of surface engineering methods like low-temperature plasma treatments is their ability to produce layers on the scale of tens of nanometers. This ensures minimal dimensional changes to implants, maintaining compatibility with standardized implantation systems. Moreover, these methods provide precise control over layer thickness through adjustments in process parameters and enable the treatment of components with complex geometries.

The materials used in orthopedics must meet standard biomaterial requirements such as biocompatibility, mechanical strength, wear resistance, and corrosion resistance to prevent metallosis—the release of alloy components into the biological environment—which can cause inflammation, allergic reactions, or even implant failure. In addition, they must fulfill specific orthopedic requirements, including high fatigue resistance to withstand repetitive mechanical loads, strong osseointegration to bond directly with bone tissue, and adequate tensile strength and flexibility to endure complex bodily movements.

The purpose of this review is to explore the recent advancements in surface modification techniques for NiTi alloys, with a particular emphasis on low-temperature plasma oxidation. This process has gained significant attention due to its ability to enhance the corrosion resistance, biocompatibility, and mechanical stability of NiTi-based implants without compromising their unique shape memory and superelastic properties. This review provides additional insights by focusing on the challenges related to scaling up these methods for industrial applications and achieving consistent reproducibility across complex geometries. Specifically, it highlights how controlling process parameters with greater precision can lead to tailored implant surfaces, potentially improving clinical outcomes. The review also highlights the practical examples of NiTi applications in orthopedics, including fracture fixation devices, spinal implants, and joint prostheses. Additionally, it discusses future perspectives, focusing on innovative surface engineering methods, such as hybrid plasma treatments and multifunctional coatings, which have the potential to further expand the clinical use of NiTi alloys in orthopedic surgery.

## 2. Biocompatibility of NiTi Alloys

The excellent biocompatibility, corrosion resistance, and unique mechanical properties of NiTi alloys make them promising materials for medical applications, with many designs already in clinical use [4,5,6]. However, their use, especially in long-term implants, is limited mainly due to their high nickel content (>50% atomic). Therefore, improving the biocompatibility of NiTi alloys remains a major research focus [7,8,9,16,20].

The biocompatibility of NiTi alloys is generally considered high, primarily due to the self-passivation effect, where a thin and stable oxide layer forms spontaneously on the surface due to titanium’s high chemical affinity for oxygen [23,24]. These passive layers provide corrosion resistance comparable to [25] or even greater than [24] that of stainless steels. However, the literature indicates that the passive layers formed on NiTi alloys are relatively thin (up to 5–8 nm) [26], and their self-healing process after damage may be slower than their dissolution in the human body environment [27,28]. As Toker et al. [29] demonstrated, the effectiveness of surface passivation—and thus the degree of biocompatibility—depends on the implant’s geometry and its implantation site in the body. Damage to the passive layer, such as from prolonged exposure to physiological fluids, intensifies nickel ion release from the surface [26]. Moreover, the naturally formed passivation layer may contain small amounts of nickel oxides (NiO and Ni_2_O_4_) and metallic nickel [30], which are more easily released than in oxide form. Notably, the rate of nickel release into the surrounding environment decreases over time [31].

Thus, the primary concern with NiTi alloy applications in medicine remains their high nickel content. Although trace amounts of nickel naturally occur in the human body, exceeding the safe daily dose (approximately 200–300 µg) can cause allergic reactions, cytotoxic effects, and even carcinogenic responses [10,11,32,33,34,35]. Chrzanowski [34] described cytoskeletal changes in osteoblasts after exposure to various concentrations of metal ions, including nickel. He found that even low concentrations (0.1–0.5 mM) of nickel, cobalt, and vanadium ions negatively affected cell responses, indicating greater cytotoxicity than elements such as aluminum, chromium, molybdenum, and iron, which only affected cells at much higher concentrations (10 mM).

Similarly, Plant [32,33] demonstrated the negative impact of nickel ions on human vascular endothelial cells, showing increased oxidative stress and impaired cell function. Wataha et al. [36] investigated the secretion of inflammatory cytokines by macrophages exposed to various metal ions, including nickel. They found no significant changes in cytokine levels (IL-1β and TNF-α) after 24 h; however, after 72 h, nickel ions significantly increased cytokine levels. Notably, inflammatory responses were triggered at concentrations where previous in vitro and in vivo studies reported metal ion release from alloys used in dentistry. Similar observations were made during the direct exposure of cells to NiTi and other biomedical alloys [37].

Therefore, increasing the biocompatibility of NiTi alloys through enhanced corrosion resistance—and thereby limiting or eliminating metallosis (ion release from the alloy surface into the biological environment)—has become crucial. Additionally, controlling cellular responses through surface modifications tailored to the intended application is essential. Shaping such biocompatibility remains one of the primary challenges in modern surface engineering.

## 3. Surface Modification Techniques for NiTi Alloys

The surface modifications of NiTi shape memory alloys aim to improve their corrosion resistance, wear resistance, and nickel isolation from the surface, thereby enhancing biocompatibility and tailoring the biological properties of the surface through controlled cellular responses. The effect of surface modifications on the properties of NiTi alloys is currently the subject of extensive research [12,13,14,15,16,17,18,20,21,24,26,38,39,40,41,42,43,44,45,46,47,48,49,50,51,52,53,54,55,56,57,58,59]. By selecting appropriate surface treatment methods and adjusting specific technological parameters, it is possible to control properties such as surface chemical and phase composition, microstructure, topography, wettability, surface energy, residual stress state, electric charge, and mechanical properties. Each of these features can directly or indirectly influence processes occurring at the material–biological environment interface, including protein adhesion and biofilm formation, cell adhesion and proliferation, the secretion of inflammatory mediators, and bacterial and microbial adhesion and growth [60]. The course of these processes determines the success or failure of biomaterial implantation.

Surface engineering plays a crucial role in expanding the application potential of biomaterials, including NiTi shape memory alloys. Various surface engineering methods are applied to tailor the properties of NiTi alloys for medical applications, including air and water passivation, ion implantation, laser treatments, electrochemical oxidation, sol–gel processes, chemical and physical vapor deposition (CVD and PVD), glow discharge treatments, and hybrid techniques. The resulting layers include oxides, nitrides, and carbon-based, oxide, polymer, or apatite coatings [8,13,16,34,57,58,59,61].

### 3.1. Air or Steam Oxidation

NiTi alloys, like other titanium alloys, spontaneously form a thin passive oxide layer primarily composed of titanium dioxide (TiO_2_) and mixed oxides within milliseconds of exposure to air [62]. Increasing the temperature can thicken this passive layer. Researchers [63] observed morphological changes and increased oxide layer thickness on NiTi alloys when heat-treated at temperatures up to 800 °C, with significant changes occurring above 300 °C.

An XPS analysis of the phase composition of mechanically polished NiTi samples revealed a passive layer mainly consisting of TiO_2_ with traces of Ti_2_O_4_ and 13.8% nickel. After thermal oxidation at 300 °C, TiO_2_ increased while metallic nickel decreased to 3.5%. Nickel hydroxide increased after oxidation at 400 °C, though no metallic nickel was detected. At 600 °C, the oxide layer consisted of 95% TiO_2_, with nickel hydroxide reduced fourfold, indicating improved nickel isolation [32,33].

Thermal oxidation at 675 °C for 30 min and 700 °C for 60 min produced TiO_2_ layers with thicknesses of 0.25 µm and 2.75 µm, respectively, containing 1.5–1.7% nickel. Pre-nitriding using gas mixtures reduced surface nickel content only when performed at 1000 °C for 20 min in a 96% N_2_ + 4% H_2_ atmosphere, suggesting that thicker TiN layers contribute to effective nickel isolation [39].

However, thick oxide layers formed at temperatures above 600 °C may be brittle, and damage could accelerate corrosion and nickel ion release due to underlying nickel-enriched zones [39]. Research on bioactivity showed apatite formation after heat treatment at 600 °C and 800 °C following incubation in simulated body fluid (SBF), indicating potential for bone implant applications [64].

Oxide layers can also be formed in boiling water or steam autoclaves, with thicknesses up to 30 nm depending on treatment time. Although reduced nickel content was observed to depths of 10 nm, nickel concentration increased with prolonged boiling [64,65]. Hydrogen peroxide (H_2_O_2_) solutions have also been used to create oxide layers, preceded by surface cleaning with Kroll’s solution. While the resulting layers isolated nickel, they exhibited poor adhesion and microcracking [40,66].

Steam autoclaving produced amorphous TiO_2_ layers about 3 nm thick after 30 min at 134 °C, with thickness controlled by processing time and temperature [67,68,69]. These layers lacked nickel but showed slight increases in nickel concentration with extended processing while retaining good adhesion and flexibility under shape recovery conditions [68].

### 3.2. Electrochemical Oxidation

Electrochemical (anodic) oxidation creates passive layers through redox reactions in an electrolyte under an applied voltage, forming oxide layers up to several hundred nanometers thick, enhancing corrosion resistance and biocompatibility [60]. Layer thickness correlates with color changes. Titanium and its alloys can be anodized using galvanostatic, potentiostatic, or combined methods, adjusting the current density, potential, process time, and electrolyte type [60]. Studies [41,70] produced 20–25 nm thick passive layers through galvanostatic anodization in 1M CH_4_COOH for 1 h at 20 mA/cm^2^. These layers were amorphous, crack-free, and showed improved corrosion resistance in Hank’s solution at 37 °C. The Ni/Ti ratio decreased from 0.30 to 0.04, although trace amounts of Ni_2_O_4_ were detected near the surface [70].

### 3.3. Ion Implantation

Initially, the ion implantation process required the treated surface to be aligned with the ion beam, making the process directional [60]. However, the introduction of Plasma Surface Ion Implantation (PSII) enabled the treatment of entire components, which is especially beneficial for complex-shaped medical implants [60]. An additional advantage of this method is that the process temperature is near room temperature, preventing any adverse impact on the mechanical properties of NiTi alloys [71]. Furthermore, the absence of a distinct boundary between the treated layer and the substrate, due to gradual chemical composition changes, enhances the stress resistance associated with shape memory effects [71].

A study [12] demonstrated oxygen ion implantation on NiTi alloys under a 50 kV voltage and an ion dose of 3 × 10^17^ ions/cm^2^. Microscopic observations and XPS analyses identified three distinct surface zones: an 800 nm thick amorphous titanium dioxide (TiO_2_) layer, a 350 nm transitional layer containing Ti_4_Ni_2_O precipitates, and a deeper layer with Ni_4_Ti_4_ precipitates. Nickel oxides such as NiO and Ni_2_O_4_ were also detected, with their depth of occurrence increasing after ion implantation compared to the untreated state [12].

Ion implantation can also create nitride layers. In one study [42], a TiN layer approximately 180–200 nm thick with an amorphous nanocrystalline structure was formed. Two sublayers with a blurred boundary were identified, containing 41% atomic nitrogen at a depth of 50 nm, decreasing gradually to about 255 nm. Nickel was present only in trace amounts up to 50 nm, with its maximum concentration observed between 75 and 100 nm.

Implanting nitrogen ions at a dose of 1 × 10^18^ ions/cm^2^ produced a 180–200 nm thick layer [42]. Nitrogen was detected up to 225 nm, while nickel was found in trace amounts up to 50 nm, with concentrations increasing at greater depths. The presence of nitride layers significantly enhanced the surface hardness (more than doubling with a nitrogen dose of 2 × 10^18^ ions/cm^2^) and wear resistance compared to untreated samples [72,73]. Nitrogen ion implantation can also be applied to complex-shaped components using plasma techniques [72,73].

Additionally, carbon-based layers can be created using plasma ion implantation. This process involves using a carbon-containing gas (e.g., acetylene C_2_H_2_), which is ionized in a radio-frequency field, allowing the carbon ions to bombard the surface and form a layer. Research [43] showed that the resulting diamond-like carbon (DLC) layers were amorphous with nanocrystalline regions. These layers exhibited good adhesion and minimal thickness, and did not affect the martensitic transformation kinetics. They also improved corrosion resistance [74], blocked nickel ion release from the samples’ surfaces [43], and demonstrated excellent hemocompatibility [74].

### 3.4. Laser Treatments

Laser treatments are becoming increasingly popular for producing oxide layers on NiTi alloys. These layers form due to the melting of the surface under high-intensity pulsed laser radiation without affecting the bulk material properties [71]. Studies [44,75] demonstrated oxide layer formation using a pulsed Nd-YAG laser with the following parameters:

- Frequency: 10 Hz;
- Pulse Duration: 9 ns;
- Pulse Energy: 400–600 mJ;
- Beam Width: 11 mm;
- Scanning Speed: 0.1 mm/s;
- Beam Divergence: 3.0 mm.

At a pulse energy of 500 mJ, a 25 nm thick passive layer was created. Increasing the scanning speed by 100 times yielded a layer thickness of 75 nm. These layers were homogeneous, crystalline, and free of visible cracks, though they exhibited increased surface roughness compared to untreated samples. Transmission electron microscopy (TEM) revealed heat-affected zones with “wave-like” features up to 60 nm deep.

Increasing laser intensity produced thicker (up to 10 µm) and more textured layers with enhanced tribological properties but reduced corrosion resistance [45]. XPS analysis showed that laser-generated oxide layers reduced the Ni/Ti ratio from 0.30 (for polished samples) to 0.17, maintaining this ratio to depths of several tens of nanometers.

Laser techniques also enable the formation of nitride layers with good adhesion. In studies [46,76], a pulsed Nd-YAG laser was used in a nitrogen atmosphere (power: 500 W, beam width: 2 mm, and scanning speed: 5 mm/s), producing a 2 µm thick TiN layer with dendritic TiN precipitates penetrating the NiTi matrix. This layer significantly increased surface microhardness, reduced surface nickel concentration to 1.5% at., and lowered nickel release rates in Hank’s solution without altering the martensitic transformation range.

### 3.5. Sol–Gel Method

The sol–gel method is advantageous for the surface modification of NiTi alloys due to its low cost and simplicity. It involves the transformation of a sol into a gel through the gradual dehydration of metal hydroxide sol. The hydrolysis reaction of metal alkoxides produces metal hydroxide and alcohol. The subsequent heating of the gel at 430–830 °C results in the formation of powders or oxide coatings [60,71]. Deposition techniques include dip-coating, spin-coating, spray-coating, or continuous dip-coating. Coatings can be single- or multi-component [60,71].

Studies [47,48,77] explored oxide coatings produced via sol–gel on NiTi alloys. Their thickness depended on dip cycles, precursor concentrations (e.g., Ti(OC_4_H_9_)_4_ and Ti(OBu)_4_), and application speed. Films up to 750 nm thick were produced. The coatings were amorphous TiO_2_ and were subjected to hydrothermal treatment in water vapor at 105 °C (24 h), 200 °C (8 h), or argon at 500 °C (1 h). Higher processing temperatures increased surface roughness, while optimal corrosion resistance was achieved at 105 °C (an increase in corrosion potential by 89 mV), while at higher temperatures, the results were only slightly better or similar to those of polished samples. The produced coatings also exhibited poor adhesion. After incubation in platelet-rich plasma (PRP), they showed good platelet adhesion, indicating their biocompatibility [48].

### 3.6. Chemical Vapor Deposition (CVD)

Chemical vapor deposition (CVD) involves processes utilizing volatile chemical compounds transported to a substrate, where a coating forms as a result of a chemical reaction [78]. An example of using CVD in modifying NiTi alloys is the production of carbon-based layers through plasma-enhanced CVD. In studies [79,80], the process was conducted at 150 °C for 5 to 20 min. Some samples were pre-passivated, creating a 3.5 nm amorphous TiO_2_ layer. The thickness of carbon layers ranged from 32 to 57 nm. These layers were amorphous with crystalline regions attributed to phases such as diamond-like carbon (DLC), TiO, or TiC. Nickel migration into the carbon layer was significantly reduced due to the presence of the thin passive layer [80]. These coatings also contributed to improved corrosion resistance [80]. Observations from these studies support the concept of combining oxidation processes with carbon layer formation

### 3.7. Physical Vapor Deposition (PVD)

Physical vapor deposition (PVD) encompasses processes conducted under reduced pressure, where coating growth occurs from vapor-phase molecules obtained through physical methods such as metal or alloy evaporation or sputtering. This process results in coatings with adhesive bonding to the substrate [78].

An example of using PVD techniques in the treatment of NiTi alloys is the IBAD (Ion-Beam-Assisted Deposition) process. This method aimed to create a carbon coating with randomly distributed silver nanoparticles on a pre-oxidized NiTi alloy [50]. The process involved sputtering a graphite plate with a centrally positioned vertical silver strip using an argon ion beam. Ejected carbon and silver atoms were directed toward the NiTi sample surface, forming an amorphous carbon coating of type a-C(Ag). The resulting hybrid layer featured an outer zone of amorphous carbon embedded with silver nanoparticles. This structure enhanced surface roughness, improved corrosion resistance, reduced wettability and surface free energy, and decreased platelet adhesion, aggregation, and activation compared to untreated NiTi alloys [50].

### 3.8. Plasma Electrolytic Oxidation (PEO)

Plasma Electrolytic Oxidation (PEO) is a surface modification technique that has garnered significant attention for enhancing the properties of NiTi alloys. PEO creates a thick, porous, and adherent oxide layer, predominantly composed of titanium dioxide (TiO_2_) in its anatase and rutile phases. This oxide layer significantly improves corrosion resistance by acting as a barrier to nickel ion release, thereby enhancing the biocompatibility of the alloy [81].

PEO processes have many advantages: they allow for the formation of thick and well-adhered oxide coatings on various metals, enhancing their hardness and wear resistance [82,83]. This makes PEO particularly valuable in applications where durability is critical, such as long-term biomedical implants. Secondly, PEO enables precise control over the composition and structure of the oxide layer, offering versatility in tailoring surface properties to meet specific functional requirements. This flexibility makes PEO a preferred choice for improving corrosion resistance and promoting biocompatibility in materials. Furthermore, the PEO process is environmentally friendly, as it typically involves water-based electrolytes and does not rely on hazardous chemicals. This aligns with the growing emphasis on sustainable and eco-friendly manufacturing processes. Although they are widespread for titanium and its alloys [84,85,86], the application for the NiTi alloy is less common. Depending on the selection of the electrolyte composition, it is possible to incorporate elements such as calcium or phosphorus into the coating structure. Moreover, thanks to the porous structure of the produced coatings, it is also possible to incorporate elements with antibacterial properties or even drugs into the coating.

However, the process requires the precise control of electrical parameters and electrolyte composition to ensure uniformity and reproducibility, which can be a challenge for scaling up in clinical applications.

### 3.9. Low-Temperature Plasma Treatments

Low-temperature plasma oxidation refers to the use of ionized gases like oxygen or air in a plasma state to create an oxide layer on a substrate at temperatures below 300 °C. This technique forms a protective or functional oxide film, enhancing corrosion resistance, biocompatibility, and surface reactivity without altering the bulk properties of the material.

Plasma can be generated by applying an electric field to a gas, causing ionization. Common methods include the following:

1. Direct Current (DC) Discharge: A constant voltage ionizes the gas between two electrodes, producing plasma.
2. Radio Frequency (RF) Discharge: An alternating current in the radio frequency range generates plasma efficiently at lower pressures.
3. Microwave Discharge: Microwaves induce plasma by transferring energy to electrons, ideal for uniform treatment.
4. Dielectric Barrier Discharge (DBD): An insulating barrier prevents continuous arcs, creating a stable, low-temperature plasma.

These methods are widely used in oxidation processes for precise surface modifications.

#### Low-Temperature Glow Discharge Plasma Treatments

In this variation, a glow discharge creates reactive oxygen species that interact with the surface, promoting a controlled process (e.g., oxidation). It ensures uniform layers with precise thickness and composition, ideal for applications like biomedical implants. Glow discharge treatments belong to a group of plasma-based techniques utilizing a specific type of electrical discharge in gases known as glow discharge, which occurs at pressures ranging from 10^−3^ to 13 hPa [78]. This process is characterized by the emission of light, commonly referred to as cathode glow. The resulting plasma contains ions, electrons, excited particles, atoms, and gas molecules, which increases the system’s energy. The high concentration of chemically active species allows processes to occur rapidly at relatively low temperatures.

In glow discharge plasma, the plasma is generated by applying an electric field between two electrodes in a low-pressure gas environment. The key steps include the following:

1. Gas Ionization: A working gas (e.g., oxygen) is introduced into the chamber.
2. Electrical Breakdown: A high-voltage electric field ionizes the gas, creating free electrons and ions.
3. Glow Discharge Formation: The energized electrons collide with gas molecules, creating a stable, glowing plasma.
4. Surface Interaction: Reactive oxygen species produced by the plasma oxidize the material surface, forming an oxide layer.

This method ensures precise oxidation while maintaining low substrate temperatures.

Glow discharge treatments offer several advantages, including the following:

1. The ability to treat components with complex shapes.
2. The capability to tailor the properties of the produced layers by controlling process parameters such as temperature, time, gas atmosphere composition, pressure in the working chamber, and current and voltage between electrodes.
3. The possibility of conducting the process at temperatures not exceeding 300 °C is crucial when treating shape memory alloys due to the need to preserve specific mechanical properties [18].

**Figure 1:**
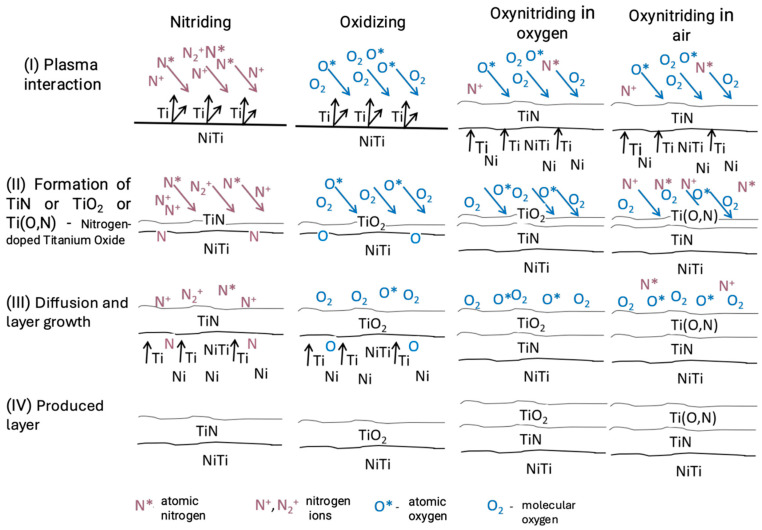
Schematic of the interaction of low-temperature glow discharge plasma with NiTi alloy depending on the applied gas atmosphere.

The layers produced through glow discharge nitriding are diffusion-based, characterized by high hardness and good adhesion to the substrate [17,71,87]. Depending on the applied gas atmosphere, nitriding, oxidation, or oxynitriding processes can be performed, combining nitriding and oxidation in a single technological cycle. The schematic of layer formation on NiTi alloy under glow discharge conditions is presented in Figure 1.

The impact of low-temperature glow discharge nitriding on the properties of NiTi alloy was investigated in a study [88]. The process was conducted at temperatures ranging from 200 to 300 °C, under a pressure of p = 4 hPa, for durations between 10 min and 1.5 h. The treatment showed a minor effect on the characteristic temperatures of the martensitic transformation and altered its behavior from a single-stage to a two-stage process. Both examined processes (nitriding and oxynitriding by glow discharge) caused a slight decrease in shape recovery and reduced the force required to induce specific deformations, indicating improved superelastic properties of the NiTi alloy.

Studies [87,89] investigated the structure of glow discharge nitrided layers formed at various temperatures using X-ray reflectometry. The thickness of the titanium nitride (TiN) layer increased with temperature. Microscopic analysis confirmed the nanocrystalline structure of the produced layers, which enhanced the corrosion resistance of the NiTi alloy. No nickel content was detected in the Tyrode’s solution used during testing.

Studies [90,91,92] demonstrated that glow discharge nitriding processes conducted at higher temperatures (700 °C and 800 °C) resulted in the formation of a brittle Ti_2_Ni intermediate layer, limiting its applicability in shape memory-related applications due to deformation constraints. Additionally, research [88] found that temperatures above 300 °C induced Ni_4_Ti_3_ precipitations, reducing the shape recovery capability of the material.

Mechanical property investigations of glow discharge oxynitrided layers [93] indicated that both hardness and reduced elastic modulus were higher for the oxynitrided layer compared to the NiTi core. Furthermore, the friction coefficient was lower for the oxynitrided NiTi alloy across all the tested loads and sliding paths. These findings suggest enhanced wear resistance after low-temperature plasma processing, which is crucial for the industrial applications of NiTi alloys.

Research [17] showed that TiN layers produced under glow discharge conditions at temperatures below 300 °C at a chamber operating pressure of 1.6 mbar exhibited a nanocrystalline structure and improved corrosion resistance compared to untreated NiTi. Additionally, these layers reduced the formation of intermetallic phases from the Ni-Ti system beneath the titanium nitride sublayer, which could negatively impact the properties of Nitinol. These layers demonstrated enhanced osteoblast proliferation, evaluated at 24 h, 48 h, and 6 days, indicating improved biocompatibility [17]. The formation of the TiN layer also resulted in increased adhesion, aggregation, and activation of blood platelets [17]. Such findings are promising for the potential use of NiTi alloys in bone implant applications.

**Figure 2:**
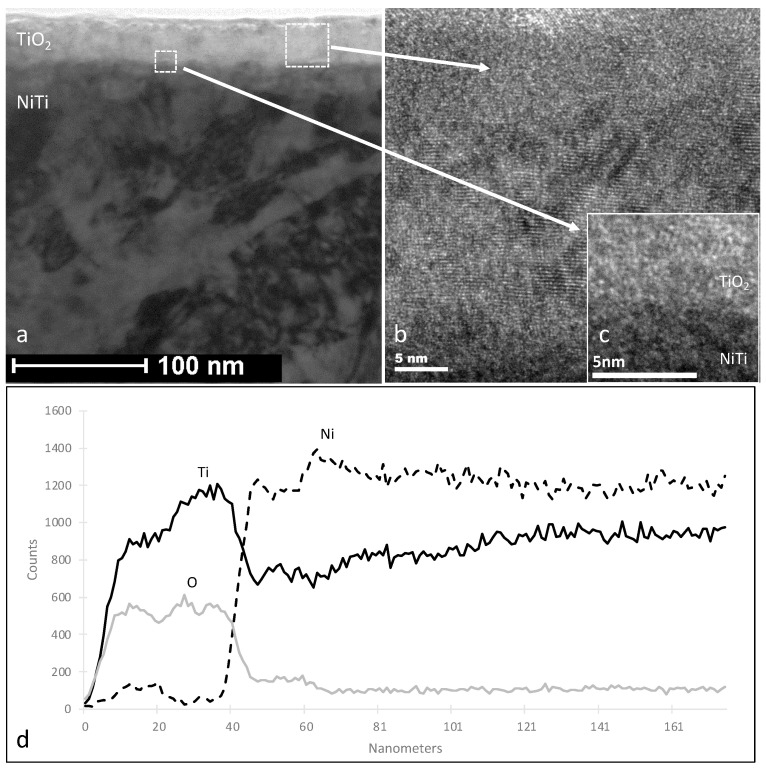
Microstructure (TEM) of titanium oxide—TiO_2_ (**a**–**c**) surface layers produced by oxidation in an oxygen atmosphere without preliminary cathode sputtering. Microstructure of the surface layer (**a**), the structure of the oxide layer (**b**), and transition zone (**c**). Distribution of titanium, nickel, and oxygen in the studied layers (**d**) Reprinted with permission from Ref. [95]. Copyright 2025 Elsevier.

It was indicated that glow discharge plasma treatments on NiTi alloys produce compact, homogeneous surface layers such as titanium dioxide (TiO_2_), a combination of TiO_2_ and titanium nitride (TiN), and nitrogen-doped TiO_2_ with TiN (Ti(O,N)+TiN) depending on the applied gas atmosphere. The thickness of these layers ranges from 20 to 40 nm [94]. Surface roughness increased after treatment. These layers also significantly improved the corrosion resistance of NiTi alloys with superior corrosion resistance exhibited by the TiO_2_ and TiO_2_+TiN layers formed under glow discharge plasma conditions. The created surface layers also significantly reduced the release of nickel ions from the samples compared to the untreated NiTi, as analyzed after a 14-day incubation in SBF solution at 37 °C using inductively coupled plasma atomic emission spectrometry (ICP-AES) [95] (Figure 2). No significant differences were observed between the types of surface layers produced, nor between other types studied with the a-C:N:H carbon coating. This suggests that the primary barrier to nickel ion release is formed by the TiO_2_ and TiO_2_+TiN layers created during glow discharge oxidation and oxynitriding processes. Corrosion resistance tests conducted using the potentiodynamic method confirmed that oxidation in low-temperature plasma improves the corrosion resistance of NiTi alloy, which can be eventually further enhanced by the addition of an amorphous carbon coating on the surface. This is evidenced by a decrease in the corrosion current density, along with an increase in the corrosion potential.

Among various methods, low-temperature plasma oxidation (LTPO) has emerged as a promising approach due to its ability to create uniform and chemically stable surface layers with enhanced functional properties.

When compared to traditional methods such as thermal oxidation or chemical treatments, LTPO offers superior control over surface composition and structure. Unlike high-temperature methods, LTPO minimizes the risk of altering the alloy’s bulk properties, such as its shape memory and superelasticity. Additionally, LTPO achieves a balance between improved corrosion resistance and bioactivity by generating biocompatible titanium dioxide (TiO_2_) layers with excellent adhesion and stability. Furthermore, the ability to incorporate nitrogen during the process (plasma-assisted oxynitriding) enables the formation of hybrid layers, such as TiO_2_+TiN, which enhance both the mechanical and chemical properties.

Despite its advantages, LTPO faces challenges related to scalability and reproducibility. The specialized equipment required for plasma generation can incur significant costs, which may limit its adoption for large-scale manufacturing. Moreover, ensuring uniform layer formation across complex geometries remains a technical hurdle. Specifically, low-temperature plasma treatments may face obstacles in achieving uniform plasma generation when applied to larger components. Variability in plasma parameters and environmental factors can also affect reproducibility, particularly when transitioning from laboratory to industrial-scale applications. Scaling up requires advanced plasma equipment capable of controlling parameters such as pressure, gas composition, and power distribution across larger substrates. Additionally, adapting these methods to industrial production lines demands process automation to ensure reproducibility and efficiency while minimizing operator intervention. Also, the costs associated with specialized equipment and extended processing times may hinder large-scale adoption for the orthopedic implant market. Addressing these challenges will involve integrating advanced diagnostics and control systems to monitor and adjust plasma conditions in real-time, ensuring consistent outcomes even at industrial scales, and is critical to fully harness the potential of LTPO in advancing surface engineering for medical devices.

### 3.10. Other Types of Coatings

Various types of coatings can be applied to modify the surface of NiTi alloys, including metallic, polymeric, and bioactive coatings.

Metallic Coatings: Metals such as tantalum, niobium, zirconium, and platinum are commonly used for surface modifications due to their high corrosion resistance and biocompatibility [71].

Polymeric Coatings: Polymeric coatings provide flexibility and corrosion resistance. In a study [51], poly(lactic acid) (PLA) was used to coat NiTi alloy strips via solution deposition at room temperature. The 2–3 µm thick PLA layer reduced surface roughness without impairing the shape memory effect during cyclic loading and unloading tests. The elastic nature of the polymer prevented cracking during mechanical testing. Other polymers used as coatings for NiTi include polypropylene (PP), polyethylene oxide (PEG), polyethyleneimine (PEI), and others [96]. When selecting polymeric materials, factors such as biodegradability must be considered, especially for long-term implants where continuous protection against metallosis is required.

Bioactive Coatings: Bioactive coatings such as hydroxyapatite are used to enhance osteointegration due to their chemical similarity to bone mineral composition. Synthetic hydroxyapatite with a calcium–phosphorus ratio similar to natural bone is the most common choice [52,71,97]. Other calcium phosphate-based materials include whitlockite (tricalcium phosphate, TCP) and biphasic calcium phosphate (BCP), combining hydroxyapatite and whitlockite [98,99]. In one study [99], calcium phosphate coatings were applied to steam-autoclaved NiTi substrates, demonstrating excellent potential for biomedical applications. Hydroxyapatite coatings showed the best biocompatibility, adhesion, and resistance to the adverse effects linked to the shape memory effect, emphasizing their suitability for orthopedic implants.

### 3.11. Hybrid Methods

Two-step, or hybrid, surface treatment methods involve the sequential application of two or more surface engineering processes to create a composite surface layer with properties unattainable by using a single surface treatment technology [78]. Examples include combining hydroxyapatite thermal spraying with heat treatment, plasma nitriding or carbonitriding with Pulsed Laser Deposition (PLD), or chemical autocatalytic deposition of nickel–phosphorus coatings with plasma treatments.

Another approach involves combining plasma nitriding and oxidation in a single technological cycle known as plasma oxynitriding or combining low-temperature glow discharge plasma oxidizing with biomimetic apatite coating formation [100], which can have great importance for orthopedic applications. So far, the relationship between the type of TiO_2_ titanium oxide and the deposition of various types of calcium phosphate on its surface has not been well described. Literature data indicate that the crystalline phases of titanium dioxide, anatase and rutile, have a good ability to induce hydroxyapatite nucleation on the surface [101,102,103,104]. It turns out that the most important aspect may be matching the crystal lattice of the titanium oxide (rutile or anatase) to hydroxyapatite, i.e., the distribution of -OH groups on the surface plays a key role, and no less important is the surface charge [105]. Some researchers speculate that anatase is characterized by higher bioactivity than rutile due to the better adjustment of its lattice to hydroxyapatite and higher acidity, as well as the lower zeta potential caused by a greater number of hydroxyl groups on the surface [106]. There are also reports of the higher bioactivity of rutile [107], as well as studies in which the mixture of rutile and anatase oxides was the most bioactive [101,108]. It is known that in addition to the crystal structure, other parameters may be important, such as the thickness of the oxide layer [109] or surface topography, especially at the nano scale [107]. There is, so far, no single mechanism that would explain all these relationships. Understanding the phenomena occurring at the interface of biomaterial surfaces and body fluids in a biological environment is a current research problem.

## 4. Titanium Oxide Layer and Its Biological Properties

Titanium is a highly reactive metal that oxidizes readily in various environments. This oxidation process occurs extremely rapidly, within the first nanoseconds of exposure [24,110]. As a result, titanium surfaces in the air are naturally covered by an oxide layer (pure metallic titanium surfaces exist only under specific conditions, such as ultra-high vacuum). In most cases, this surface layer primarily consists of titanium dioxide (TiO_2_), the most stable form of titanium oxide. Unlike titanium, TiO_2_ is a chemically stable compound, resistant to most chemical agents. The excellent chemical and corrosion resistance of titanium is largely attributed to the chemical stability of the surface titanium dioxide layer [24,111].

**Figure 3:**
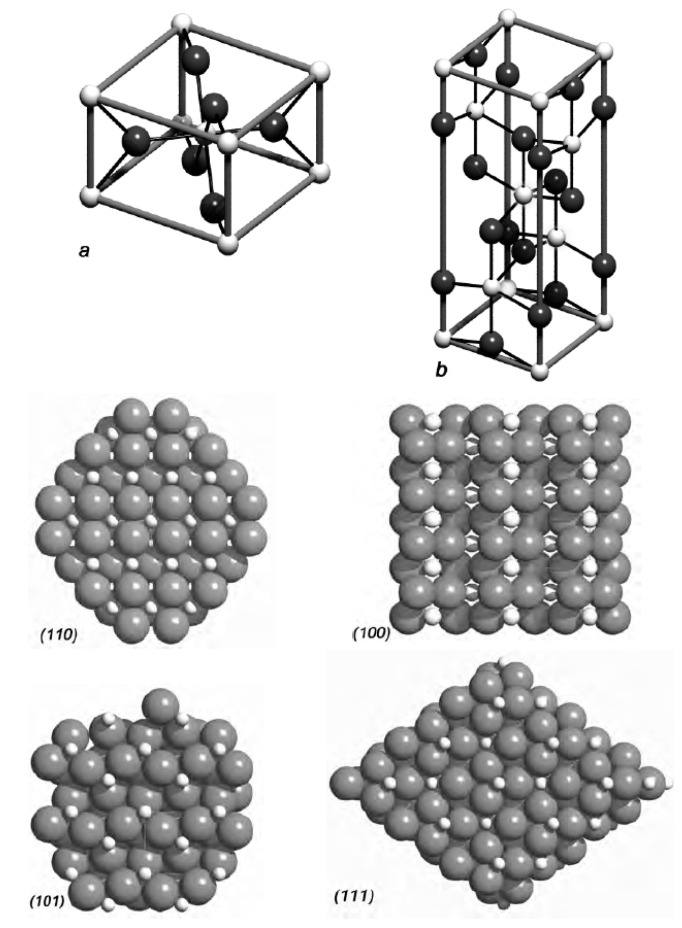
Unit cells of the two most common titanium dioxide (TiO_2_) polymorphs: rutile (**a**) and anatase (**b**). Below: ideal single-crystal rutile surface structures with orientations (110), (100), (101), and (111). Small white spheres = titanium cations; large gray spheres = oxygen anions. Reprinted with permission from Ref. [24]. Copyright 2025 Springer Nature.

Titanium dioxide (TiO_2_) exists in three different crystallographic forms: rutile, anatase, and brookite. Rutile and anatase have a tetragonal structure, while brookite has an orthorhombic structure. In these structures, titanium atoms are surrounded by six oxygen atoms forming octahedra. The arrangement of these octahedra differs among the three forms. Figure 3 illustrates the unit cells of rutile (a) and anatase (b), along with the views of four single-crystal rutile surfaces, assuming ideal planar cuts through the rutile structure at specific angles [24].

The unit cell parameters are as follows:

Rutile: a = 0.459 nm, b = 0.459 nm, and c = 0.295 nm;

Anatase: a = 0.378 nm, b = 0.378 nm, and c = 0.951 nm;

Brookite: a = 0.544 nm, b = 0.917 nm, and c = 0.514 nm.

From an application perspective, rutile and anatase are the most important forms of TiO_2_, with rutile being the most thermodynamically stable polymorph [24,112].

The transition from anatase to rutile occurs at approximately 600 °C, although this temperature can change due to various factors, such as the presence of impurities or small amounts of brookite, which can accelerate the transition and lower its temperature [113,114,115]. Pure titanium dioxide (TiO_2_) is an n-type semiconductor, with different band gap energies for its polymorphic forms: 3.23 eV for anatase, 3.02 eV for rutile, and 2.96 eV for brookite [116]. Titanium dioxide also exhibits an exceptionally high refractive index (higher than that of diamond), with values of approximately 2.9 for rutile, 2.6 for anatase, and 2.8 for brookite [112].

**Figure 4:**
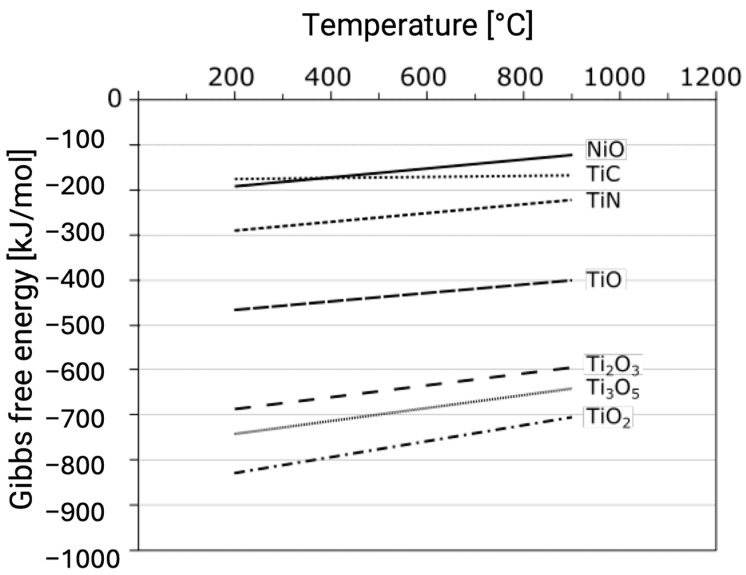
The Gibbs free energy of formation for titanium oxides, titanium nitride (TiN), titanium carbide (TiC), and nickel oxide (NiO) (based on [117]).

According to thermodynamic principles, Gibbs free energy favors the formation of TiO_2_ over nickel oxides or other titanium oxides (Figure 4) [117]. Additionally, studies on the Ti6Al4V alloy have demonstrated that rutile forms under glow discharge conditions, even when titanium nitride undergoes oxidation [19,118].

The naturally forming titanium oxide layer on titanium and its alloys are primarily responsible for their superior corrosion resistance and biocompatibility compared to other materials. This layer significantly influences interactions at the implant–biological environment interface, playing a critical role in the body’s biological response to the implanted biomaterial [24,53,60].

The biological properties of titanium dioxide (TiO_2_) are promising for medical applications. Literature data indicate that titanium oxide layers promote osteoblast adhesion and proliferation [119,120], making them suitable for long-term orthopedic and dental implants [120]. After implantation, bone tissue remodeling and osteointegration occur, establishing a direct connection between the live bone tissue and the titanium implant surface [60,103,119,121]. Additionally, titanium oxide can facilitate the adsorption of calcium ions and phosphate ions (PO_4_^3−^) from physiological solutions due to its negatively charged surface in aqueous environments [62,122,123,124]. This promotes the formation of natural apatite, further enhancing biocompatibility and accelerating osteointegration. Studies show that this process occurs more rapidly with crystalline structures [60] and materials with higher surface roughness [103]. Titanium oxide also demonstrates good biocompatibility with fibroblasts [53] and neurons [125] and antibacterial properties [126].

## 5. Clinical Relevance and Applications of NiTi Alloys

Oxidized nickel–titanium (NiTi) alloys have garnered significant attention in the medical field due to their enhanced biocompatibility and mechanical properties. The formation of a titanium dioxide (TiO_2_) layer on NiTi surfaces plays a pivotal role in improving corrosion resistance and reducing nickel ion release, thereby enhancing patient safety.

The clinical advantages of oxidized NiTi implants include the following:

○ Reduced nickel ion Release and enhanced patient safety: The TiO_2_ layer acts as a protective barrier, significantly minimizing the release of nickel ions, which is crucial given the potential cytotoxic and allergenic effects of nickel. This reduction is vital for ensuring the biocompatibility of NiTi implants [127].
○ Improved bioactivity promoting osteointegration: The oxidized surface facilitates better integration with bone tissue. Studies have shown that TiO_2_ layers enhance the adhesion and proliferation of osteoblasts, leading to improved osteointegration [61,119,128,129].
○ Enhanced antibacterial properties reducing the risk of infection: The TiO_2_ layer exhibits antibacterial properties, which help in reducing the risk of postoperative infections, a critical factor in the success of implant surgeries [126].

Applications of NiTi in orthopedics include the following:

○ Joint Prostheses: Oxidized NiTi alloys can be potentially utilized in hip, knee, and shoulder implants due to their superelasticity and shape memory properties, which allow for better conformity and durability [130]
○ Spinal Implants: The flexibility and strength of oxidized NiTi make it suitable for spinal applications, including rods, screws, and cages, providing enhanced support and alignment [130,131]
○ Trauma Fixation Devices: In trauma cases, oxidized NiTi plates, screws, and intramedullary nails offer superior fixation and adaptability to complex fracture patterns [132].

Among the various biomedical applications, solutions enabling total knee replacement (TKR) have become one of the most frequently discussed in orthopedics, which results from a growing number of replacement and revision surgeries [133,134]. The TKR implant consists of three main elements: the femoral component, the tibial component (the tibial tray and the tibial insert), and the patellar component [135]. The tibial insert and the artificial patella are usually made of plastics, such as ultra-high-molecular-weight polyethylene (UHMWPE) or crosslinked polyethylene (XLPE), and the femoral component and tibial tray are usually made of metals including Ti-based alloys, stainless steels, and Co–Cr–Mo alloys or sometimes they are fabricated from ceramics such as alumina, zirconia, and their composites. These components can be cemented (by polymethylmethacrylate or PMMA) or cement-free—which is a more modern approach. However, it requires the use of modern biomaterials and an understanding of the mechanisms of natural knee loading [135].

Various material solutions for TKR implants are currently available on the market, among which the most popular are proposed by the following companies: Zimmer Biomet, Stryker, DePuy Synthes, and Smith & Nephew. They are based on the previously mentioned materials, i.e., Co–Cr alloys, Ti alloys, and plastics such as UMWPE or PA. Despite their popularity, they still do not meet all the requirements at once and cause various postoperative complications. It is estimated that in 20% of patients, total knee arthroplasty does not achieve satisfactory results [136]. Some of them suffer from implanted metal hypersensitivity, and this group of patients in particular needs new implants with biocompatible coatings [56,137]. Therefore, there is still a search for better and better solutions based on other, new materials that minimize the risk of reoperation and increase patient comfort and safety.

The most common problems that cause complications after knee arthroplasty and the need for revision surgery are as follows:

1. The aseptic loosening of the implant due to excessive wear of the joint surfaces (particles that are products of wear—mainly UHMWPE—activate cellular processes that cause osteolysis, i.e., bone resorption associated with foreign body response [138];
2. The effect of stress shielding of the bone by the prosthesis, resulting mainly from properties of material (modulus of elasticity), causing gradual osteolysis [139];
3. The development of fibrous tissue at the interface between bone and implant [137].

Considering these problems and the geometry of the knee prosthesis design, the selection of the optimal metal alloy for the femoral and tibial parts of the implant plays a significant role in extending the life of TKR implants and preventing revision surgeries. A list of requirements for biomaterials considered for knee implants has been proposed in the literature, together with the consequences of not fulfilling the requirements (Table 1) [140].

Traditionally, the choice of new material or the replacement of the existing material with other properties that provide better performance was usually carried out using trial and error methods or following previous experience. However, the material selection process can be optimized by using the multiple criteria decision-making (MCDM) models [141] to avoid the misuse of materials, which involves huge costs. MCDM provides the basis for selecting and prioritizing when choosing materials, and assists in the overall assessment. Recently, it has been applied to the problem of the material solution of the TKR implant, taking into account such criteria as strength, Young’s modulus, ductility, corrosion resistance, wear resistance, and susceptibility to osseointegration. The simulations indicated that the most promising material is NiTi shape memory alloys (the porous NiTi alloy came first followed by a dense NiTi alloy). Currently used materials, such as stainless steels, CoCr alloys, or other titanium alloys, are only found in further places [135]. It should be emphasized that the concept of porous titanium oxide on the surface of a dense NiTi alloy will be a better alternative to the use of a porous NiTi alloy due to the better corrosion resistance of such a solution. In the case of a porous alloy, a much larger surface area of the alloy has contact with the biological environment. Although it is indicated as the number one among the most promising materials for a knee implant [135], it should be taken into account that this simulation did not include, as a criterion, even corrosion resistance or the limitation of the release of Ni ions from the surface.

During everyday activities, such as walking, climbing stairs, or lifting various objects, the human body, in particular the skeletal part, is exposed to stress. It can cause deformation in the prosthetic joints, and that is why it is so important that after the end of the procedure, the implant returns to its designated shape. Therefore, the superelastic properties of the NiTi shape memory alloy can contribute to extending the implant’s lifetime (no irreversible or residual deformations) [135]. In addition, the low Young’s modulus of this alloy will also reduce the maximum pressure on the implant–bone interface, and thus the material wear rate [142], and minimize the effect of stress shielding [143]. However, the slow process of osteogenesis, as well as the production of fibrous tissue at the interface of NiTi alloy implants with bone, leading to the poor binding of the prosthesis to the surrounding bone and implant micro-movements, make it necessary to apply surface modifications increasing its bioactivity. Hence, the production of thin layers of apatite on NiTi alloy implants is the hope of increasing and accelerating osseointegration [144]. However, due to the porous structure and poor adhesion to the substrate [61], they do not provide sufficient protection against metallosis, which can be ensured by the earlier oxidation of the alloy. So, the latest trends in material engineering development, including surface engineering, can improve the properties of materials using hybrid technologies.

Some specific orthopedic applications of surface-modified NiTi alloys have already been introduced into clinical practice and are the subject of extensive research [145,146,147,148]. For instance, hydroxyapatite (HA)-coated and silanized NiTi implants have demonstrated enhanced performance and significant potential for orthopedic applications in vivo [149]. Similarly, a study evaluating surface-treated NiTi orthodontic archwires, including Titanol Low Force River Finish Gold (Forestadent, Pforzheim, Germany), showed that these wires initially exhibited significantly lower friction compared to non-treated wires [150]. Comparable findings were observed regarding the corrosion resistance of the wires, indicating that surface-treated NiTi wires exhibit improved initial performance, which may degrade over time during clinical use [151]. Exploring these functional aspects is crucial, as NiTi alloys, despite their high resistance to corrosion fatigue, have shown susceptibility to microcracking under cyclic loading in corrosive environments [152,153]. The clinical significance of these findings remains underexplored, with limited long-term studies validating these solutions in real-world applications [6]. Addressing these challenges through advanced, potentially hybrid, surface engineering methods and testing the new solutions in environments mimicking real-world conditions is critical to unlocking the full potential of NiTi alloys in biomedical applications. Companies such as Zimmer Biomet have introduced the NiTi UNITUS™ Staple System, which exhibits enhanced fatigue strength, measured at four times more cycles at a 50% higher load compared to a leading competitive staple [154]. Medtronic incorporated NiTi spinal staples into their CD Horizon™ Spinal System, intended to provide the immobilization and stabilization of spinal segments [155]. Additionally, NuVasive Inc. launched the NuVasive Thoracolumbar Plates system, a lateral and anterolateral plating solution comprising plates, bolts, screws, and related surgical instruments, which includes NiTi elements [156]. These advancements illustrate that surface engineering techniques can enable the successful integration of NiTi alloys into diverse orthopedic applications, driving improved clinical outcomes.

## 6. Limitations of NiTi for Orthopedic Implants

The main criticisms of NiTi alloys for orthopedic implants are centered on material, biological, and mechanical concerns. NiTi alloys contain about 50% nickel, which raises concerns regarding toxic effects, allergic reactions, and inflammatory responses. Although oxide layers can mitigate nickel ion release, implant degradation over time may lead to cytotoxic and genotoxic effects. In a biological environment, NiTi implants are vulnerable to corrosion fatigue due to mechanical stress and a corrosive medium. Without protective coatings, surface wear can expose nickel-rich layers, compounding the risk of ion release. While NiTi exhibits good fatigue resistance, long-term cyclic loading can result in implant failure. The shape memory effect, though advantageous, can be unpredictable without precise control, complicating implant deployment and design. NiTi is inherently bioinert, requiring surface modifications like hydroxyapatite coatings or plasma oxidation to enhance bioactivity and bone integration. However, these advanced treatments add significant production costs.

Despite promising clinical results, limited long-term data exist to validate NiTi implants’ safety and efficacy. Additionally, the nickel content complicates regulatory approval processes in certain regions with stringent biocompatibility standards. NiTi’s unique properties necessitate precise processing and heat treatments, making manufacturing challenging and expensive compared to materials like stainless steel or cobalt–chromium. Efforts to address these issues include advanced surface coatings, improved corrosion resistance, optimized implant designs, and expanded long-term clinical research.

## 7. Conclusions and Future Perspective

NiTi alloys have demonstrated remarkable potential for orthopedic applications due to their unique superelasticity, shape memory effect, and mechanical properties. However, their clinical use remains constrained by issues such as nickel ion release, limited bioactivity, and corrosion susceptibility. Advances in surface modification techniques, particularly low-temperature plasma oxidation (LTPO), have shown significant promise in overcoming these limitations. LTPO facilitates the formation of uniform, nanocrystalline TiO_2_ layers and hybrid TiO_2_-TiN coatings, which enhance corrosion resistance, improve biocompatibility, and promote bioactivity while preserving the alloy’s intrinsic mechanical properties. Despite these advancements, further efforts are needed to optimize LTPO for broader clinical applications. Future research should focus on integrating hybrid surface treatments, combining bioactive coatings or antibacterial functionalities to further improve osseointegration and reduce infection risks. Additionally, long-term clinical studies are essential to validate the durability and safety of modified NiTi implants in real-world medical settings. Addressing the challenges related to scalability and cost-efficiency through advancements in plasma processing technology will be crucial for the widespread adoption of these surface-modified implants. Furthermore, the integration of drug delivery systems and customized implant designs, enabled by additive manufacturing technologies, could significantly enhance the functionality and patient-specific applicability of NiTi implants. Regulatory considerations will also play a critical role in bringing modified NiTi alloys to clinical use. Surface-modified NiTi implants must comply with stringent biocompatibility, corrosion resistance, and mechanical stability standards as outlined by organizations such as the U.S. Food and Drug Administration (FDA) and the European Medicines Agency (EMA). Key steps in the regulatory pathway include preclinical testing to evaluate cytotoxicity, genotoxicity, and in vivo biocompatibility, as well as mechanical testing under simulated physiological conditions. Clinical trials will be essential to establish safety and efficacy, with a focus on long-term outcomes such as corrosion resistance, nickel ion release, and implant integration. By refining these technologies and ensuring clinical validation, surface-modified NiTi alloys hold the potential to revolutionize orthopedic and biomedical applications, delivering safer, more durable, and effective solutions for patients.

## Figures and Tables

**Table 1:** Requirements of biomaterials and problems derived from inadequate requirements [140].

Significant Requirements	Consequences of Not Fulfilling the Requirements
Long fatigue life	Implant mechanical failure and revision surgery
Adequate strength	Implant failure, pain to patient, and revision surgery
Modulus equivalent to that of bone	Stress shielding effect, loosening, failure, and revision surgery
High wear resistance	Implant loosening, severe inflammatory response, and destruction of the healthy bone; producing wear debris that can go to the blood
High corrosion resistance	Releasing noncompatible metallic ions and allergic reactions
Biocompatibility	Body reaction and adverse effects in the organic system
Osseointegration	Fibrous tissue between the bone and the implant, improper integration of the bone and implant, and finally, implant loosening

## Data Availability

Data are available upon request from the corresponding author (the data are not publicly available due to technical and organizational restrictions).
